# Dissociable roles of the hippocampus and parietal cortex in processing of coordinate and categorical spatial information

**DOI:** 10.3389/fnhum.2014.00073

**Published:** 2014-02-17

**Authors:** Oliver Baumann, Jason B. Mattingley

**Affiliations:** ^1^Queensland Brain Institute, The University of Queensland, St. LuciaQLD, Australia; ^2^School of Psychology, The University of Queensland, St. LuciaQLD, Australia

**Keywords:** categorical, coordinate, spatial, memory, navigation, fMRI, hippocampus, parietal

## Abstract

It is generally accepted that spatial relationships and spatial information are critically involved in the formation of cognitive maps. It remains unclear, however, which properties of the world are explicitly encoded and how these properties might contribute to the formation of such maps. It has been proposed that spatial relations are encoded either categorically, such that the relative positions of objects are defined in prepositional terms; or as visual coordinates, such that the precise distances between objects are represented. Emerging evidence from human and animal studies suggests that distinct neural circuits might underlie categorical and coordinate representations of object locations during active spatial navigation. Here we review evidence for the hypothesis that the hippocampal formation is crucial for encoding coordinate information, whereas the parietal cortex is crucial for encoding categorical spatial information. Our short review provides a novel view regarding the functions and potential interactions of these two regions during active spatial navigation.

## Introduction

As humans navigate they build up mental models of the physical world, which are indispensable for finding one’s way in complex environments and planning routes to distant locations. Previous human and animal studies have suggested that mental models of spatial environments are not maintained as a unitary representation; instead, different aspects of space appear to be underpinned by many different cognitive subsystems and brain regions (e.g., Bohbot et al., 2000; Epstein and Higgins, 2007; Zhang et al., 2012; Baumann and Mattingley, 2013). A prominent example of this representational fragmentation is the distinction between *egocentric* and *allocentric* representations of object locations. The egocentric representational system is thought to provide transient action-oriented representations of the environment from the viewpoint of the navigator (for an overview see reviews by Klatzky, 1998; Burgess, 2006). Several lines of evidence indicate that egocentric representations are supported by the parietal lobe. For example, lesions of the posterior parietal cortex are known to disrupt patients’ ability to point to the locations of objects in the absence of visual input (Levine et al., 1985), and neurons in the intraparietal sulcus have been found to respond to visual and auditory targets in a body-centered fashion (Mullette-Gillman et al., 2005). In contrast, the allocentric system is thought to provide a comprehensive and enduring representation of the environment that is accessible from any viewpoint (Klatzky, 1998; Burgess, 2006). In humans and rodents, the hippocampus has been found to contain view-invariant cells that fire selectively as a function of an animal’s location in space, but show little dependence on the animal’s egocentric orientation during active navigation (O’Keefe and Dostrovsky, 1971; Ekstrom et al., 2003).

## Categorical vs. coordinate spatial representations

Another hypothesized dichotomy within spatial memory is the distinction between categorical and coordinate representations of space. Categorical spatial relationships capture general properties of the spatial layout, referring to broad equivalence classes of spatial positions relative to reference stimuli (e.g., left/right, below/above, inside/outside). By contrast, coordinate spatial relationships refer to precise spatial locations, which can be expressed in terms of metric units between locations (e.g., Object A is located 2.4 m from Object B). This distinction was originally proposed by Kosslyn (Kosslyn, 1987; Kosslyn et al., 1989, 1992), based on neural network simulations that showed that it is computationally more efficient to represent categorical and coordinate spatial information separately. Kosslyn also made anatomic predictions, proposing that the left hemisphere is involved specifically in processing categorical information, whereas the right hemisphere is involved in processing coordinate information. According to his original theory, such a hemispheric asymmetry might have arisen for two reasons. First, the left hemisphere advantage for categorical processing could have emerged from its pre-existing dominance for language, particularly with respect to category formation, whereas the right hemisphere advantage for coordinate processing could have arisen from its fundamental role in spatial navigation (Kosslyn, 1987; Kosslyn et al., 1989). In this context, however, it is important to mention that the left hemisphere advantage for categorical processing has also been observed in monkeys (Jason et al., 1984; Vogels et al., 1994) and pigeons (Yamazaki et al., 2007), implying that language is not the only factor that aids category formation. An alternative explanation for the hemispheric asymmetry is that it might arise from a difference in the receptive field properties of neurons in the two hemispheres (Kosslyn et al., 1992; Jacobs et al., 1994; Chabris and Kosslyn, 1998). According to this account, the right hemisphere has a bias to encode outputs from neurons with relatively large receptive fields, whereas the left hemisphere has a bias for neurons with relatively small receptive fields. The assumption is that non-overlapping receptive fields divide space into simple categorical relations, whereas large, overlapping receptive fields support the encoding of precise coordinate relations. This hypothesis is corroborated by the observation that the left hemisphere is biased to process signals from the parvocellular visual pathway, whereas the right hemisphere is biased to process signals from the magnocellular visual pathway (Kosslyn et al., 1992; Roth and Hellige, 1998; Hellige and Cumberland, 2001).

## Distinct neural networks underlie encoding of categorical and coordinate spatial relations

**Figure 1:**
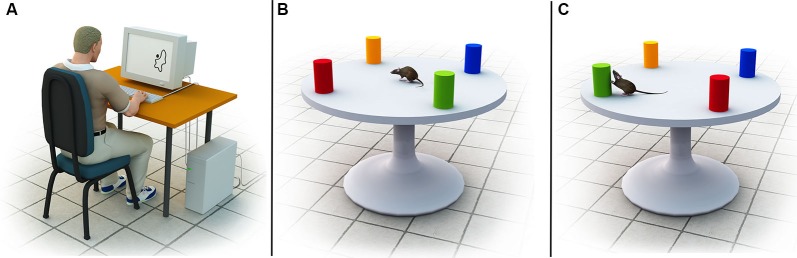
**(A)** Example of the stimuli used in the experiment by Kosslyn et al. (1989). The stimuli were amorphous outline figures with a large dot located 0, 1 or 10 mm from the border of the figure. Participants in the coordinate task were asked to judge whether the dot was within 2 mm of the contour of the blob, whereas participants in the categorical task were asked to judge whether the dot was on or off the contour. **(B)** Illustration of the spatial environments used by Goodrich-Hunsaker et al. (2005). Shown is the geometric configuration of colored objects on top of a round board. **(C)** After rats habituated to the environment, either a categorical or coordinate transformation was applied (in this illustration, a categorical transformation via a left-right transposition of the green and red landmarks is shown). Subsequent assessment of the rats’ re-exploration behavior served as an indicator of the precision of their internal representation of the layout.

Despite the uncertainty regarding a theoretical explanation, several human studies have provided experimental evidence for the hypothesized separation of coordinate and categorical representations of space. Evidence has come from three main sources: (1) visual half-field studies in healthy participants; (2) neuroimaging investigations; and (3) behavioral studies in patients with brain lesions. The proposed hemispheric lateralization effect has been most consistently observed in the parietal cortex (cf. Jager and Postma, 2003), but has also been detected in frontal areas (Slotnick and Moo, 2006; van der Ham et al., 2009). It is important to add, however, that the evidence for hemispheric specialization originates almost exclusively from experiments in which human participants are asked to encode and recall visual stimuli within static, two-dimensional displays (cf. Jager and Postma, 2003). A classic example is the seminal study by Kosslyn et al., 1989; see Figure 1A) in which participants either judged whether a dot was on or off the contour of a line drawing of a nonsense shape (the categorical task), or whether the dot was within 2 mm of the contour (the metric task). Later studies replicated the hemispheric specialization effect using more realistic stimuli, such as meaningful objects (Saneyoshi et al., 2006) and natural scenes (van der Ham et al., 2011). These studies, however, did not examine whether the distinction between coordinate and categorical representations would also apply to the encoding of three-dimensional spatial environments, in which individuals must build up a representation based upon continually changing visual inputs obtained from a first-person perspective.

Initial evidence for separate coordinate and categorical representations of three-dimensional spatial environments was obtained in animals. Goodrich-Hunsaker et al. (2005) employed a novelty-detection task in brain-lesioned rats to investigate the neural circuits underlying the encoding of categorical and coordinate spatial relations during active navigation. Rats were first familiarized with a geometric object configuration on top of a round board (see Figure 1B). Subsequently, either the categorical or coordinate relationship between the geometric objects was altered (e.g., via a left-right transposition or distance change), and the rats’ exploration behavior was recorded and assessed as an indicator of their spatial knowledge (see Figure 1C). Rats naturally familiarize themselves with their environment via initial exploration (*habituation*, Poucet, 1993), and if a change in the environment occurs they typically spend substantially more time re-exploring the environment (*dishabituation*, Save et al., 1992). The ratio of the total time spent exploring the displaced objects and the total time spent exploring the non-displaced objects can be used as an indicator of the reliability of the underlying spatial representation. For example, if a rat spends a significant amount of time re-exploring two objects that have undergone a categorical transformation, but fails to respond to a coordinate transformation, it can be presumed that the rat has a compromised ability to encode coordinate relations, whereas its ability to encode categorical relations is unimpaired. Using this approach, Goodrich-Hunsaker et al. (2005) discovered that rats with dorsal hippocampal lesions displayed deficits in coordinate spatial learning tasks, but behaved normally in categorical tasks. On the other hand, rats with parietal lesions showed significant impairments in categorical spatial memory tasks but not in coordinate tasks.

Thus, whereas the human literature has suggested a hemispheric specialization (left vs. right) for the encoding of categorical and coordinate spatial relations, the rodent data imply a structural specialization (parietal cortex vs. hippocampus). It is important to note, however, that the relevant human studies have employed almost exclusively static, two-dimensional stimulus arrays, whereas the rodent studies have used three-dimensional mazes and arenas that the animal is required to learn through active exploration. A possible explanation for the apparent discrepancy between the human and rodent research, therefore, is that the two species process categorical and coordinate information differently, or that the discrepancy is due to the different paradigms used in people and rats.

**Figure 2:**
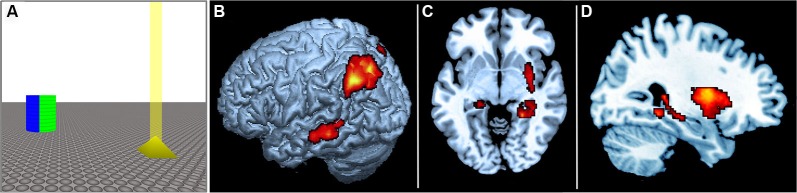
**(A)** Schematic of the virtual environment used in the experiment by Baumann et al. (2012). The blue and green side of the reference landmark is shown. The target is shown in yellow, with a virtual “beacon” projecting vertically from its apex. Participants were required to actively navigate the arena and to encode either the distance of the target relative to the landmark (coordinate task), or the sector in which the target object was located (categorical task). The two task conditions employed visually identical virtual environments and differed only in the instructions to the participants (i.e., coordinate task: “Remember the distance”; categorical task: “Remember the sector”). **(B)** Rendered image of left hemisphere showing corresponding fMRI data. **(C)** Axial view. **(D)** Sagittal view. There was greater left posterior parietal activity during the encoding of categorical spatial relations, and greater right hippocampal activity during the encoding of coordinate spatial relations.

We recently investigated this question by using fMRI to monitor brain activity while human participants actively navigated a three-dimensional virtual arena, which was similar to those employed in rodent research (Baumann et al., 2012). The virtual arena consisted of an infinite plane and contained just two geometric objects, with one serving as a landmark and the other as a target. The landmark was a cylinder rendered in four different colors, virtually dividing the arena into four quadrants, and the target was a small yellow pyramid (see Figure 2A). On every trial, the participants were required to locate and navigate to the target object using a hand-held joystick. In the categorical condition, participants were instructed to remember the *quadrant* in which the target object was located, as defined by the color-code of the central landmark. By contrast, in the coordinate condition participants were instructed to remember the *distance* between the target object and the landmark, irrespective of the quadrant in which the target was located. After a brief maintenance period participants re-entered the virtual environment, but the target object was now absent. In the coordinate condition participants were required to navigate back to the remembered *distance* of the target object from the cylindrical landmark, ignoring the quadrant, whereas in the categorical condition participants were required to navigate back to the remembered *sector* of the target’s location, irrespective their distance from the central landmark. To determine whether distinct neural substrates are responsible for the encoding of categorical and coordinate aspects of spatial environments, encoding-related fMRI activity was compared using a simple subtraction approach. The results revealed a hemispheric as well as structural dissociation for categorical vs. coordinate memory encoding. In line with previous studies in humans, the categorical condition led to predominantly left hemispheric activity, whereas the coordinate condition activated primarily the right hemisphere. Moreover, in line with the relevant rodent literature, categorical encoding led to stronger activity in the parietal cortex, whereas coordinate encoding led to stronger activity in the hippocampus (see Figures 2B–D).

## Hippocampal and parietal contributions to spatial-relation coding

The notion that the hippocampus underlies the encoding of coordinate spatial relations is in line with earlier human imaging studies, which showed that the hippocampus is typically active whenever spatial associations have to be formed in a way that allows for absolute metric accuracy during navigation. A recent example is an fMRI study in which we observed a close relationship between hippocampal activity and metric accuracy in a memory-guided navigation task (Baumann et al., 2010). We measured neural responses as participants learned the location of a single target object relative to a small set of landmarks. Following a delay, the target was removed and participants were required to navigate back to its original position. We found that greater activity in the right hippocampus during object-location encoding predicted higher metric accuracy in locating the hidden target object in the retrieval phase. Other human imaging studies have implicated the human hippocampus even more explicitly in the encoding of distance information. Morgan et al. (2011) recorded brain activity using fMRI while university students were shown images of landmarks from a familiar college campus. They found that activity in the hippocampus scaled with the distances between landmarks. More specifically, the hippocampal response to each landmark was dependent on the real-world distance between that landmark and the landmark shown on the preceding trial. This distance-related effect was observed in the absence of any explicit navigational task—participants were simply asked to think about the identity of each landmark—suggesting that this process operates automatically. Further evidence for a hippocampal role in distance coding comes from a recent imaging study by Viard et al. (2011), in which participants were asked to indicate the shortest egocentric distance to a target location from varying locations in a virtual environment. The hippocampus showed a very robust increase in activation with goal proximity, in line with its hypothesized role in encoding coordinate properties of the spatial environment.

While crucial for the encoding of coordinate spatial relations, lesion data in rodents suggest that categorical spatial relations can be encoded in the absence of an intact hippocampus (Goodrich-Hunsaker et al., 2005). Lesions of the parietal lobe, however, have been found to cause severe deficits in rodents during such tasks, suggesting an important role for this structure in the encoding of categorical spatial relations. In humans, the posterior parietal cortex has long been known to play a pivotal role in the short-term maintenance of spatial information (for a meta-analysis, see Wager et al., 2003). However, several lines of evidence also point to a more specific role of this structure in categorical spatial information processing. Early evidence came from clinical reports, which suggested that lesions of the posterior parietal lobe can lead to left-right (Laeng, 1994) or inside-outside confusion for locations in space (Robertson et al., 1997). In a later study, Ciaramelli et al. (2010) reported that patients with lesions in the posterior parietal cortex show deficits in making landmark sequence (i.e., categorical) judgments, but are unimpaired in distance and proximity (i.e., coordinate) judgments. In recent years, a series of neuroimaging studies have provided additional evidence for a role of the human parietal cortex in encoding categorical spatial relations (cf. Jager and Postma, 2003). An interesting example is a study by Amorapanth et al. (2010), which found that having participants direct their attention to categorical spatial relations between objects, as opposed to the identity of objects, resulted in greater activity in superior and inferior parietal cortices. Finally, while most of the human findings have been based on static two-dimensional stimulus arrays, we recently demonstrated that the posterior parietal cortex is also engaged when categorical relations have to be encoded within dynamic, three-dimensional environments (Baumann et al., 2012).

## Reference frame processing vs. spatial relation coding

As mentioned above, another hypothesized dichotomy in spatial memory is the distinction between egocentric and allocentric representations of object locations. Previous studies have indicated that allocentric representations are underpinned by the hippocampus, whereas egocentric representations rely on the parietal cortex (cf. Burgess, 2006). This raises the important question of how these findings might best be integrated with the observed role of the same brain structures in coordinate and categorical spatial relation coding. Jager and Postma (2003) proposed two opposing hypotheses concerning this question. The *interaction* *hypothesis* states that allocentric processing is associated with categorical coding of spatial relations, whereas egocentric processing is closely linked to coordinate coding. The logic behind this hypothesis is that allocentric representations provide an observer with a sense of “space constancy”, defined as the awareness of relative, categorical locations of objects, which underlies an observer’s ability to recognize scenes. Coordinate representations, on the other hand, are used for action-oriented, body-centered tasks. The *interaction hypothesis* therefore predicts that categorical spatial processing should be more efficient within an allocentric reference frame, whereas coordinate processing should benefit from an egocentric reference frame. In contrast, the *independence hypothesis* states that reference frame processing and spatial relation coding form independent dimensions, which can be fully combined without showing selective facilitation.

Ruotolo et al. (2011) tested the interaction and independence hypotheses in a behavioral experiment. Participants were asked to judge the position of two vertical bars placed above and below a horizontal bar, in relation either to their body midline (egocentric reference frame) or to the center of the horizontal bar (allocentric reference frame). Moreover, they had to make distance (coordinate) judgments or relative categorical judgments. Participants were more accurate in judging categorical than coordinate relations, and especially so in the allocentric condition. This study supports the *interaction hypothesis*, suggesting that reference frame processing and spatial relation coding are not completely independent cognitive mechanisms. It is important to note, however, that the effects observed in the study of Ruotolo et al. (2011) could be task-dependent. Previous studies have shown that static, two-dimensional perceptual tasks might favor allocentric and categorical representations, whereas egocentric and coordinate information could be more relevant in three-dimensional, action-oriented tasks (cf. Schenk and McIntosh, 2010).

To answer the question whether and how reference frame processing and spatial relation coding interact, it will be necessary to determine the neural correlates of these cognitive processes in one common experiment. Only by carefully controlling both allocentric/egocentric and categorical/coordinate aspects of spatial navigation tasks will it be possible to accurately discern their relative contributions to parietal and hippocampal activation patterns. Based on current evidence, we hypothesize that spatial relation coding and reference frame processing are independent cognitive mechanisms that engage different subregions of the hippocampus and posterior parietal cortex. An allocentric-coordinate task should therefore be entirely hippocampal dependent, whereas an egocentric-categorical task would be solely dependent on the parietal cortex. On the other hand, allocentric-categorical and egocentric-coordinate navigation tasks should rely on both the hippocampus and the parietal cortex. Future experiments will be necessary to test these predictions and to provide a more mechanistic understanding of the roles hippocampal and parietal structures play in spatial relation coding and reference frame processing.

## Conclusion

There is considerable evidence for dissociable roles of the hippocampus and parietal cortex in encoding of coordinate and categorical spatial information in three-dimensional environments. Crucially, recent findings suggest that the neural networks that subserve categorical and coordinate encoding are different from those commonly reported in studies involving static, two-dimensional stimulus arrays. Given the growing body of literature suggesting a diversity of functions of hippocampal and parietal subregions (e.g., Howard et al., 2013; Poppenk et al., 2013) it will be necessary to characterize more precisely the neural foundations of categorical and coordinate encoding of spatial environments. The nature of the representations underlying human spatial cognition is still the subject of intense debate (Burgess, 2006). We believe the notion of distinct categorical and coordinate representations of spatial environments constitutes an important additional factor to be considered in building a comprehensive model of human and animal spatial navigation, and that it could have important applications beyond the laboratory. It should be possible, for example, to develop more refined behavioral tests in patients with topographical disorientation, by incorporating measures of categorical and coordinate spatial performance (Aguirre and D’Esposito, 1999).

## Conflict of interest statement

The authors declare that the research was conducted in the absence of any commercial or financial relationships that could be construed as a potential conflict of interest.

## References

[B1] Aguirre G. K. D’Esposito M. (1999). Topographical disorientation: a synthesis and taxonomy. Brain 122, 1613–1628 10.1093/brain/122.9.1613 10468502

[B44] Amorapanth P. X. Widick P. Chatterjee A. (2010). The neural basis for spatial relations. J. Cogn. Neurosci. 22, 1739–1753 10.1162/jocn.2009.21322 19642889 PMC2933471

[B2] Baumann O. Chan E. Mattingley J. B. (2010). Dissociable neural circuits for encoding and retrieval of object locations during active navigation in humans. Neuroimage 49, 2816–2825 10.1016/j.neuroimage.2009.10.021 19837178

[B3] Baumann O. Chan E. Mattingley J. B. (2012). Distinct neural networks underlie encoding of categorical versus coordinate spatial relations during active navigation. Neuroimage 60, 1630–1637 10.1016/j.neuroimage.2012.01.089 22300811

[B4] Baumann O. Mattingley J. B. (2013). Dissociable representations of environmental size and complexity in the human hippocampus. J. Neurosci. 33, 10526–10533 10.1523/jneurosci.0350-13.2013 23785164 PMC6618588

[B5] Bohbot V. D. Allen J. J. Nadel L. (2000). Memory deficits characterized by patterns of lesions to the hippocampus and parahippocampal cortex. Ann. N Y Acad. Sci. 911, 355–368 10.1111/j.1749-6632.2000.tb06737.x 10911885

[B7] Burgess N. (2006). Spatial memory: how egocentric and allocentric combine. Trends Cogn. Sci. 10, 551–557 10.1016/j.tics.2006.10.005 17071127

[B8] Chabris C. F. Kosslyn S. M. (1998). How do the cerebral hemispheres contribute to encoding spatial relations. Curr. Dir. Psychol. Sci. 7, 8–14 10.1111/1467-8721.ep11521809

[B9] Ciaramelli E. Rosenbaum R. S. Solcz S. Levine B. Moscovitch M. (2010). Mental space travel: damage to posterior parietal cortex prevents egocentric navigation and reexperiencing of remote spatial memories. J. Exp. Psychol. Learn. Mem. Cogn. 36, 619–634 10.1037/a0019181 20438261

[B10] Ekstrom A. D. Kahana M. J. Caplan J. B. Fields T. A. Isham E. A. Newman E. L. (2003). Cellular networks underlying human spatial navigation. Nature 425, 184–188 10.1038/nature01964 12968182

[B11] Epstein R. A. Higgins J. S. (2007). Differential parahippocampal and retrosplenial involvement in three types of visual scene recognition. Cereb. Cortex 17, 1680–1693 10.1093/cercor/bhl079 16997905

[B12] Goodrich-Hunsaker N. J. Hunsaker M. R. Kesner R. P. (2005). Dissociating the role of the parietal cortex and dorsal hippocampus for spatial information processing. Behav. Neurosci. 119, 1307–1315 10.1037/0735-7044.119.5.1307 16300437

[B13] Hellige J. B. Cumberland N. (2001). Categorical and coordinate spatial processing: more on contributions of the transient/magnocellular visual system. Brain Cogn. 45, 155–163 10.1006/brcg.2000.1233 11237364

[B14] Howard L. R. Kumaran D. Ólafsdóttir H. F. Spiers H. J. (2013). Dissociation between dorsal and ventral posterior parietal cortical responses to incidental changes in natural scenes. PLoS One 8:e67988 10.1371/journal.pone.0067988 23874482 PMC3706617

[B43] Jacobs R. A. Kosslyn S. M. (1994). Encoding shape and spatial relations: the role of receptive field size in coordinating complementary representations. Cogn. Sci. 18, 361–386

[B15] Jager G. Postma A. (2003). On the hemispheric specialization for categorical and coordinate spatial relations: a review of the current evidence. Neuropsychologia 41, 504–515 10.1016/s0028-3932(02)00086-6 12559166

[B16] Jason G. W. Cowey A. Weiskrantz L. (1984). Hemispheric asymmetry for a visuospatial task in monkeys. Neuropsychologia 22, 777–784 10.1016/0028-3932(84)90102-7 6527767

[B17] Klatzky R. L. (1998). “Allocentric and egocentric spatial representations: definitions, distinctions and interconnections,” in Spatial Cognition. An Interdisciplinary Approach to Representing and Processing Spatial Knowledge, eds Freksa C. Habel C. Wender K. F. (Berlin: Springer), 1–17

[B18] Kosslyn S. M. (1987). Seeing and imagining in the cerebral hemispheres: a computational approach. Psychol. Rev. 94, 148–175 3575583

[B19] Kosslyn S. M. Chabris C. F. Marsolek C. J. Koenig O. (1992). Categorical versus coordinate spatial relations: computational analyses and computer simulations. J. Exp. Psychol. Hum. Percept. Perform. 18, 562–577 10.1037//0096-1523.18.2.562 1593235

[B20] Kosslyn S. M. Koenig O. Barret A. Cave C. B. Tang J. Gabrieli J. D. E. (1989). Evidence for two types of spatial representations: hemispheric specialization for categorical and coordinate relations. J. Exp. Psychol. Hum. Percept. Perform. 15, 723–735 10.1037/0096-1523.15.4.723 2531207

[B21] Laeng B. (1994). Lateralization of categorical and coordinate spatial functions: a study of unilateral stroke patients. J. Cogn. Neurosci. 6, 189–203 10.1162/jocn.1994.6.3.189 23964971

[B22] Levine D. N. Warach J. Farah M. J. (1985). Two visual systems in mental imagery: dissociation of ‘what’ and ‘where’ in imagery disorders due to bilateral posterior cerebral lesions. Neurology 35, 1010–1018 10.1212/wnl.35.7.1010 4010939

[B23] Morgan L. K. MacEvoy S. P. Aguirre G. K. Epstein R. A. (2011). Distances between realworld locations are represented in the human hippocampus. J. Neurosci. 31, 1238–1245 10.1523/jneurosci.4667-10.2011 21273408 PMC3074276

[B24] Mullette-Gillman O. A. Cohen Y. E. Groh J. M. (2005). Eye-centered, head-centered and complex coding of visual and auditory targets in the intraparietal sulcus. J. Neurophysiol. 94, 2331–2352 10.1152/jn.00021.2005 15843485

[B25] O’Keefe J. Dostrovsky J. (1971). The hippocampus as a spatial map. Preliminary evidence from unit activity in the freely-moving rat. Brain Res. 34, 171–175 10.1016/0006-8993(71)90358-1 5124915

[B26] Poppenk J. Evensmoen H. R. Moscovitch M. Nadel L. (2013). Long-axis specialization of the human hippocampus. Trends Cogn. Sci. 17, 230–240 10.1016/j.tics.2013.03.005 23597720

[B27] Poucet B. (1993). Spatial cognitive maps in animals: new hypotheses on their structure and neural mechanisms. Psychol. Rev. 100, 163–182 10.1037/0033-295x.100.2.163 8483980

[B28] Robertson L. Treisman A. Friedman-Hill S. Grabowecky M. (1997). The interaction of spatial and object pathways: evidence from Balint’s syndrome. J. Cogn. Neurosci. 9, 295–317 10.1162/jocn.1997.9.3.295 23965009

[B29] Roth E. C. Hellige J. B. (1998). Spatial processing and hemispheric asymmetry. Contributions of the transient/magnocellular visual system. J. Cogn. Neurosci. 10, 472–484 10.1162/089892998562889 9712677

[B30] Ruotolo F. van der Ham I. J. Iachini T. Postma A. (2011). The relationship between allocentric and egocentric frames of reference and categorical and coordinate spatial information processing. Q. J. Exp. Psychol. (Hove) 64, 1138–1156 10.1080/17470218.2010.539700 21271464

[B31] Saneyoshi A. Kaminaga T. Michimata C. (2006). Hemispheric processing of categorical/metric properties in object recognition. Neuroreport 17, 517–521 10.1097/01.wnr.0000209009.70975.4c 16543817

[B6] Save E. Poucet B. Foreman N. Buhot M. C. (1992). Object exploration and reactions to spatial and nonspatial changes in hooded rats following damage to parietal cortex or hippocampal formation. Behav. Neurosci. 106, 447–456 10.1037/0735-7044.106.3.447 1616611

[B32] Schenk T. McIntosh R. D. (2010). Do we have independent visual streams for perception and action? Cogn. Neurosci. 1, 52–62 10.1080/17588920903388950 24168245

[B33] Slotnick S. D. Moo L. R. (2006). Prefrontal cortex hemispheric specialization for categorical and coordinate visual spatial memory. Neuropsychologia 44, 1560–1568 10.1016/j.neuropsychologia.2006.01.018 16516248

[B34] van der Ham I. J. Raemaekers M. Van Wezel R. J. A. Oleksiak A. Postma A. (2009). Categorical and coordinate spatial relations in working memory: an fMRI study. Brain Res. 1297, 70–79 10.1016/j.brainres.2009.07.088 19651111

[B35] van der Ham I. J. Zandvoort M. J. Frijns C. J. Kappelle L. J. Postma A. (2011). Hemispheric differences in spatial relation processing in a scene perception task: a neuropsychological study. Neuropsychologia 49, 999–1005 10.1016/j.neuropsychologia.2011.02.024 21356223

[B36] Viard A. Doeller C. F. Hartley T. Bird C. M. Burgess N. (2011). Anterior hippocampus and goal-directed spatial decision making. J. Neurosci. 31, 4613–4621 10.1523/jneurosci.4640-10.2011 21430161 PMC6622909

[B37] Vogels R. Saunders R. C. Orban G. A. (1994). Hemispheric lateralization in rhesus monkeys can be task-dependent. Neuropsychologia 32, 425–438 10.1016/0028-3932(94)90088-4 8047250

[B42] Wager T. D. Smith E. E. (2003). Neuroimaging studies of working memory: a meta analysis. Cogn. Affect. Behav. Neurosci. 3, 255–274 10.3758/cabn.3.4.255 15040547

[B38] Yamazaki Y. Aust U. Huber L. Hausmann M. Gunturkun O. (2007). Lateralized cognition: asymmetrical and complementary strategies of pigeons during discrimination of the “human concept”. Cognition 104, 315–344 10.1016/j.cognition.2006.07.004 16905127

[B45] Zhang H. Copara M. Ekstrom A. D. (2012). Differential recruitment of brain networks following route and cartographic map learning of spatial environments. PLoS One 7:e44886 10.1371/journal.pone.0044886 23028661 PMC3445610

